# Thermal conductivity engineering of bulk and one-dimensional Si-Ge nanoarchitectures

**DOI:** 10.1080/14686996.2017.1288065

**Published:** 2017-03-13

**Authors:** Ali Kandemir, Ayberk Ozden, Tahir Cagin, Cem Sevik

**Affiliations:** ^a^ Department of Materials Science and Engineering, Izmir Institute of Technology, Izmir, Turkey.; ^b^ Faculty of Engineering, Department of Material Science and Engineering, Anadolu University, Turkey.; ^c^ Department of Materials Science and Engineering, Texas A & M University, College Station, TX, USA.; ^d^ Artie McFerrin Department of Chemical Engineering, Texas A & M University, College Station, TX, USA.; ^e^ Faculty of Engineering, Department of Mechanical Engineering, Anadolu University, Eskisehir, Turkey.

**Keywords:** Molecular dynamics, thermal conductivity, superlattices, alloy, interface roughness, nanowire, thermoelectric

## Abstract

Various theoretical and experimental methods are utilized to investigate the thermal conductivity of nanostructured materials; this is a critical parameter to increase performance of thermoelectric devices. Among these methods, equilibrium molecular dynamics (EMD) is an accurate technique to predict lattice thermal conductivity. In this study, by means of systematic EMD simulations, thermal conductivity of bulk Si-Ge structures (pristine, alloy and superlattice) and their nanostructured one dimensional forms with square and circular cross-section geometries (asymmetric and symmetric) are calculated for different crystallographic directions. A comprehensive temperature analysis is evaluated for selected structures as well. The results show that one-dimensional structures are superior candidates in terms of their low lattice thermal conductivity and thermal conductivity tunability by nanostructuring, such as by diameter modulation, interface roughness, periodicity and number of interfaces. We find that thermal conductivity decreases with smaller diameters or cross section areas. Furthermore, interface roughness decreases thermal conductivity with a profound impact. Moreover, we predicted that there is a specific periodicity that gives minimum thermal conductivity in symmetric superlattice structures. The decreasing thermal conductivity is due to the reducing phonon movement in the system due to the effect of the number of interfaces that determine regimes of ballistic and wave transport phenomena. In some nanostructures, such as nanowire superlattices, thermal conductivity of the Si/Ge system can be reduced to nearly twice that of an amorphous silicon thermal conductivity. Additionally, it is found that one crystal orientation, <100>, is better than the <111> crystal orientation in one-dimensional and bulk SiGe systems. Our results clearly point out the importance of lattice thermal conductivity engineering in bulk and nanostructures to produce high-performance thermoelectric materials.

## Graphical Abstract

**Figure:**
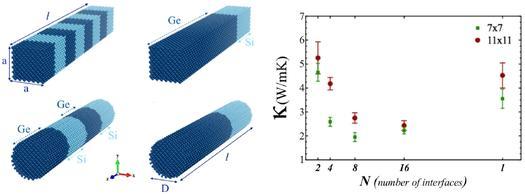

## 1. Introduction

There is an ongoing interest in low-dimensional materials, to understand transport phenomena and to realize next-generation thermoelectrics, due to the potential of these materials to contribute to a sustainable future. The interconnected performance parameters of thermoelectric materials need to be understood to determine the level of this contribution. The performance parameters of thermoelectric materials can be revealed by a dimensionless figure of merit, *ZT*(=S${}^{2}\sigma$T/($\kappa_{e}$+$\kappa_{L}$)), where S is the Seebeck coefficient, σ is the electrical conductivity, T is the absolute temperature, and $\kappa_{e}$ and $\kappa_{L}$ are the electronic and lattice thermal conductivities, respectively. Development of the *ZT* coefficient may lead to the development of devices and applications with less energy consumption. There are two mainways to increase *ZT* of low dimensional thermoelectric materials: enhancing the power factor (S${}^{2}\sigma$) [1–5] or reducing the lattice thermal conductivity without suppressing the electrical conductivity (or power factor)[6–9]. Therefore, many theoretical and experimental studies have been carried out in order to investigate the thermal transport properties of low-dimensional thermoelectric materials.

Si and Ge have been proposed as the best candidate materials for low-dimensional thermoelectric materials (nanocrystals, nanocomposites, nanowires and superlattices) due to the mature technology behind the fabrication of these nanostructures. Several experimental studies have been performed to demonstrate thermal conductivity dependence on the epitaxy, size (diameter, length), periodicity and composition of these structures. For example, epitaxial embedded Ge nanocrystals in Si have been formed by the ultrathin SiO${}_{2}$ film technique, and exhibited lower thermal conductivities (1.2 W m${}^{-1}$ K${}^{-1}$) than those of the conventional nanostructured SiGe bulk alloys with a minor reduction in electrical conductivity [10,11]. Reduced thermal conductivity in alloyed Si and Ge nanowires have been measured by Kim et al. [12]. Martinez et al. [13] fabricated 100–300 nm Si${}_{1-x}$Ge${}_{x}$ nanowires and reported 1.1 W m${}^{-1}$ K${}^{-1}$ thermal conductivity, which is close to the amorphous limit of silicon. *ZT* measurement on the same nanowires have demonstrated 0.18 times bigger *ZT* than its bulk form. Nanowires that were fabricated by Hochbaum et al. [9] with rough surfaces resulted in a *ZT* of 0.6. Moreover, it has also been reported that the nanostructured and alloyed bulk form also indicates an improved *ZT* coefficient at high temperatures, where it reaches 1.3 [14]. Thermal conductivity measurements of thin films of Si-Ge systems and superlattices have led to a renewed interest in superlattices and dimensionality problem in these systems [15–17]. For example, molecular beam epitaxy (MBE) grown Si/Si${}_{0.7}$Ge${}_{0.3}$ superlattices with periods 4.5–30 nm have demonstrated period thickness dependency where it reaches alloy limit at 4.5 nm [17]. Moreover, Si-Ge superlattice nanowires grown by Li et al. [18] have demonstrated lower thermal conductivities than 2D systems and even below the Si/Si${}_{0.9}$Ge${}_{0.1}$ alloy films, which has been attributed to the additional scattering of long wavelength acoustic phonons at the nanowire boundaries reflecting importance of dimensionality over the control of thermal transport in these systems.

Thermal transport properties and increase of the thermoelectric performance have been examined by many theoretical works for Si/Ge systems [19–29]. Si-coated Ge nanowires exhibited a significant reduction in the thermal conductivity found as revealed by non-equilibrium molecular dynamics (NEMD) [30]. Hu et al. [31,32] predicted a 75% reduction in thermal conductivity for Ge-coated Si core–shell structures with respect to the pure silicon. Mass disorder causing increased anharmonic scattering and thus decreased thermal conductivity has been investigated in Si-Ge alloys with density functional perturbation theory by Garg et al. [33]. NEMD [34,35] and EMD [36] studies have indicated lower thermal conductivity values for the ordered nanocomposite systems of Si and Ge with respect to alloys due to interface effects. Furthermore, superlattices have been proved as theoretically vital for reaching minimum lattice thermal conductivity and increasing thermoelectric performance. First, Dames and Chen [37] reported that the thermal conductivity of Si/Ge superlattice nanowires can be reduced more than two times compared to conventional superlattices. By the Monte Carlo method, Savic et al. [38] performed calculations of planar, nanowire and nanodot superlattice structures and discussed the elements of a good design for an application requiring low thermal conductivity. Using the EMD method, Haskins et al. [39] calculated the thermal conductivity of Si/Ge superlattices with quantum dots. They predicted thermal conductivity values below amorphous thermal conductivity limit and a tenfold larger *ZT* value compared to alloys or 100-fold larger compared to bulk. Landry et al. [40] studied roughness and periodicity effect in their NEMD simulations for alloy and superlattice structures of Si/Ge. They underlined the interface quality that designates the properties of a superlattice. Beside the Si/Ge core–shell calculations, via NEMD, Hu et al. [41] found that thermal conductivity of Si/Ge supercells could decrease to 10 times lower than the silicon nanowires. They remarked that periodicity optimized supercell structures can be strong candidates for thermoelectric material. *ZT* values larger than 2.0 have been reported for some of the Si-Ge nanostructures [42].

In this work, we systematically investigate the thermal conductivity of different architectures such as bulk, superlattice and alloys of silicon and germanium by using equilibrium molecular dynamics simulations. Different crystal orientations, cross-section geometries, superlattice symmetries, temperature effects, and cross-sectional properties such as interface roughness between layers are studied in detail to understand effect of nanostructuring on the thermal conductivity.

## 2. Methods

EMD simulations were performed to calculate the thermal conductivity of superlattice structures via the LAMMPS software package [43]. Thermal conductivity was evaluated by using mean square displacement of the energy moment (Einstein relation [44]). MD calculations in (NVE) microcanonical ensemble were performed with a time step of 0.5 fs. The systems first relaxed for 500 ps. Each thermal conductivity data point was obtained from the average of 10 simulations, all lasting a minimum of 4 ns. In order to accurately determine the lattice thermal conductivity of a material with molecular dynamic simulations, the Tersoff [45] potential is used; this was previously used to predict the thermal conductivity of many 1-D systems, such as Si nanowires with amorphous shell [46], Si/Ge superlattice nanowires [41,47], and Si/Ge core–shell systems [48,49]. For the lattice thermal conductivity, the Einstein relation can be written as:

(1) $$\begin{array}{cc} \kappa_{\mu \mu } & =\frac{1}{Vk_{b}T^{2}}\;\lim_{t\to \infty }\frac{1}{2t}\langle {\left[R_{\mu }\left(t\right)-R_{\mu }\left(0\right)\right]}^{2}\rangle \end{array}$$

where T, V, t, and *k${}_{b}$* are temperature, volume, time and Boltzmann constant, respectively. *R${}_{\mu }$* is the time integration of heat current in direction μ. Thus, thermal conductivity can be calculated easily with Equation (1).

**Figure 1.**
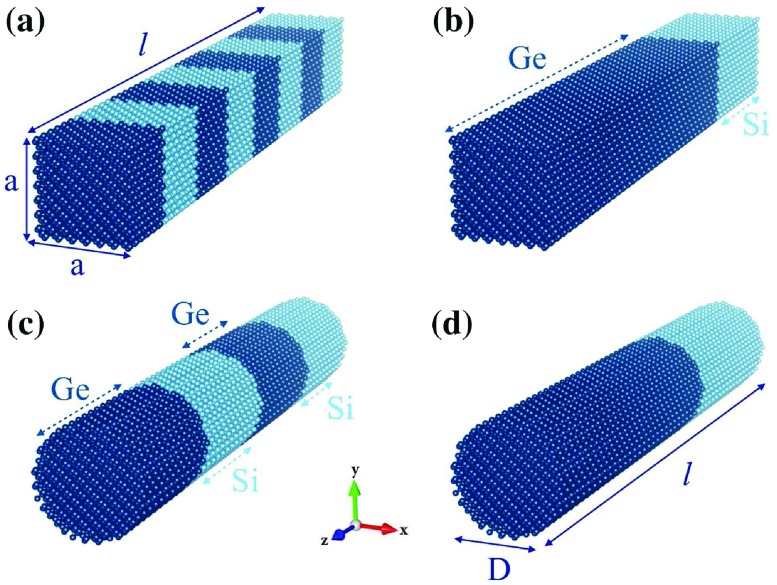
Schematic representation of (a) rectangular symmetric superlattice nanowires (periodic boundary condition is set only for z direction) and (b) asymmetric rectangular superlattices nanowires (periodic boundary condition is set only for z direction). Note that same structures of (a) and (b) represent bulk system when periodic boundary conditions are employed in x, y and z directions. (c) Symmetric cylindrical superlattice nanowires and (d) asymmetric cylindrical superlattice nanowires. Since it is not possible to apply periodic boundary conditions in x and y directions, cylindrical systems are considered as nanowires

All structures used in this study are prepared after performing relaxation simulation. Firstly, structures are created with rational lattice parameters. Second, we relax the systems and control the lattice parameters of structures at relaxation state. Structures are constructed by using the relaxed lattice parameter. Two different superlattices are considered in this study in terms of symmetry. The first one is the symmetric superlattice, which defines that silicon and germanium unit cells (UC) are equal and in sequence in the whole length of the structure. The second one is the asymmetric superlattice, which means that silicon and germanium unit cells are unequal in the whole length of the structure. Cylindrical and rectangular cross-section geometries are considered for both symmetric and asymmetric superlattices. A periodic boundary condition is applied to the growth direction to retain superlattice phenomena. Examples of symmetric and asymmetric superlattice structures are shown in Figure 1(a), (c) and 1(b), (d), respectively.

**Figure 2.**
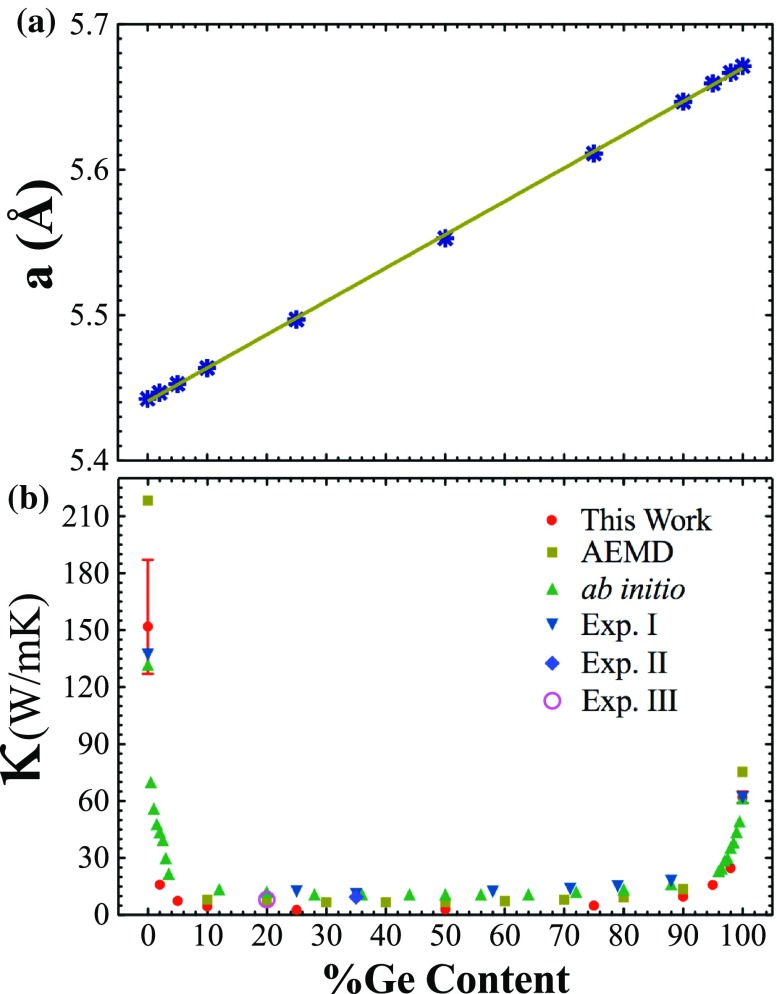
(a) Variation of lattice parameters of Si${}_{1-x}$Ge${}_{x}$ and (b) thermal conductivity values of Si${}_{1-x}$Ge${}_{x}$ with respect to Ge content.

## 3. Results

In order to confirm the accuracy of the used interatomic potential (Tersoff) and EMD parameters (relaxation time, time sampling), calculations are started with a well-known system, Si${}_{1-x}$Ge${}_{x}$ alloy. We first consider a cubic diamond structure as 12×12×12 periodic UC${}^{3}$, and alloy for *x* = 0.00 (Pure Si), 0.02, 0.05, 0.10, 0.25, 0.50, 0.75, 0.90, 0.95, 0.98 and 1.00 (Pure Ge). Figure 2 shows the calculated lattice parameters of Si${}_{1-x}$Ge${}_{x}$ after relaxation. Relaxed lattice parameters are compatible with the literature and suit Vegard’s law. Relaxed lattice parameters in Figure 2(a) are used to construct alloy structures to compute their thermal conductivity. Figure 2(b) shows thermal conductivity values of bulk alloys of this work and other experimental [14,50,51] and theoretical studies [33,52]. Our thermal conductivity data are in the same order as the other experimental and theoretical works. Moreover, the potential we use in our calculations accurately determines the Si-Ge interaction.

**Figure 3.**
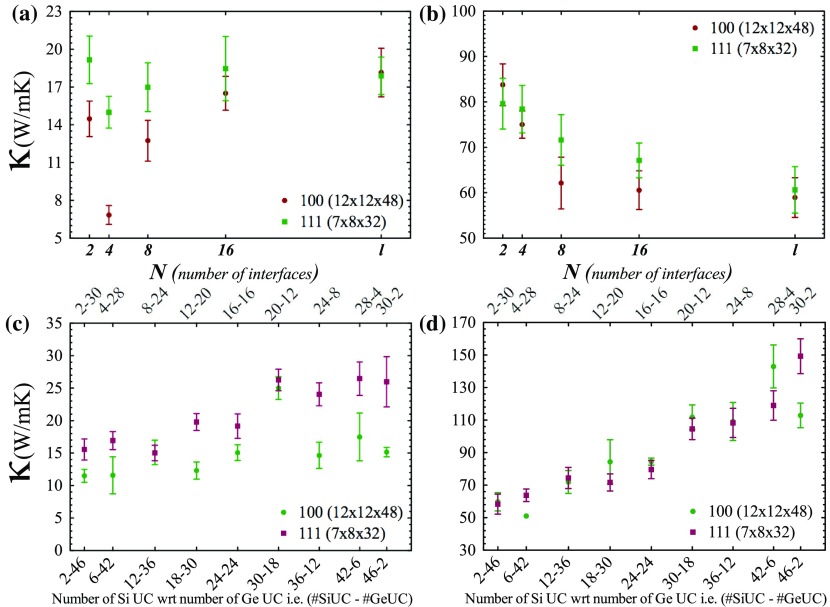
Thermal conductivity values (300 K) of symmetric superlattices with respect to number of interfaces in (a) growth direction and (b) transverse directions to growth direction. Thermal conductivity values (300 K) of asymmetric superlattices with respect to numbers of silicon unit cell comparing germanium unit cell in (c) growth direction and (d) transverse directions.

### 3.1. Bulk Si-Ge superlattices

In this part we show the effect of periodicity in two bulk superlattice structures (symmetric and asymmetric superlattices) extended in the <001> and <111> directions. Structures of 12×12 UC${}^{2}$ (∼ 42.5 nm${}^{2}$ square cross section area) with a length of 48 UC (∼ 26.5 nm) in the <001> direction and of 7×8 UC${}^{2}$-pagination (∼ 30 nm${}^{2}$ square cross section area) with a length of 32 UC (∼ 30.5 nm) in the <111> direction are investigated. To calculate the bulk lattice thermal conductivity of these structures, all directions are set as periodic.

Figure 3(a) shows the thermal conductivity along the long axis of the symmetric Si-Ge superlattice (crystallographic directions are given in the legend of Figure 3) and Figure 3(b) shows the average thermal conductivity of transverse directions with respect to the long axis. To illustrate our results, the following notation is used: periodicity is described by a parameter *w* that is calculated as *l* / *N*, where *l* is the length of the structure as unit cell and *N* is denominator of *l* and this denominator *N* can be considered as the number of interface per a supercell or nanowire. The numbers of interfaces are selected as 2, 4, 8, 16, *l* (length of supercell as UC) for investigation of thermal transport properties of symmetric Si-Ge superlattices. Therefore, the definition of periodicity (*w*) in this study is the number of unit cells of Si and Ge (always kept equal) composing one period of the superlattice. For example, a 48 UC long (*l* = 48) superlattice structure with *N* = 8 (*w* = 6) means that one period of superlattice comprise 6 UC of Si and 6 UC of Ge (Figure 1(a)). *N* = 4 for the same structure has a period consisting of 12 UC of Si and 12 UC of Ge (Figure 1(c)). Therefore, as the *N* gets larger, period thickness decreases and the number of interfaces increases. Figure 3(b) shows anticipated behavior of thermal conductivity that decreases with increasing number of interfaces due to scattering at interfaces. In other words, increase in periodicity causes the ascent of thermal conductivity on transverse directions. However, there is an anomalous behavior in growth direction (Figure 3(a)) [41]. Thermal conductivity does not follow a decreasing trend as the numbers of interfaces increases. Therefore, there is no direct relation between thermal conductivity and periodicity. We find that *w* = 8 in the <111> direction and *w* = 12 in the <001> direction correspond to the minimum thermal conductivity, and both periodicity values coincide with *N* = 4. In terms of bulk case, this coincidence and resulting minimum thermal conductivity may indicate specific length scales. In the next calculations, the same behavior is observed, so we will discuss this anomaly later in detail.

**Figure 4.**
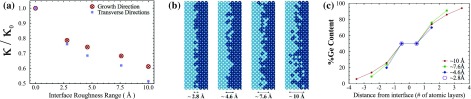
(a) Interface roughness effect on thermal conductivity; (b) schematic representation of roughness with diffusion zones changing between 0.28 and 1 nm; (c) variation of germanium content with roughness along the interface, where x=0 corresponds to the smooth pristine interface.

Next, asymmetric silicon–germanium superlattices are investigated. An asymmetric superlattice has only two interfaces in its supercell. Its description relies on the numbers of silicon and germanium UCs, which were selected as 2-46, 6-42, 12-36, 18-30, 24-24 (from symmetric superlattices), 30-18, 36-12, 42-6 and 46-2. Two of the asymmetric superlattice structures, 12-36 (12 UC Si and 36 UC Ge) and 18-30 (18 UC-Si and 30 UC Ge) are given as example structures in Figure 1(b) and (d), respectively. In those types of structure, direction dependency of thermal conductivity is revealed (Figure 3(c)). Germanium or silicon content affects thermal conductivity in the <111> direction as Figure 3(c) indicates. Thermal conductivity increases while germanium content decreases. On the other hand, along the <100> direction, thermal conductivity demonstrates a band-like behavior with values between 10 and 15 W m${}^{-1}$ K${}^{-1}$ depending on germanium content. At the point of 30 UC Si–18 UC Ge, there is peak in thermal conductivity. This peak can be associated with mean free path of low frequency phonons. In addition, there is a linear trend on transverse directions (Figure 3(d)) with respect to growth axis. Unlike alloying, controllable thermal conductivity seems possible on transverse directions with changing silicon to germanium ratio.

We also simulated the effect of interface roughness on these bulk structures. To create an interface roughness at boundaries, we used the Fick’s law of diffusion (Figure 4(c)). Figure 4(c) shows germanium content changing at different diffusion zone lengths which vary from 0.28 nm (two atomic layers) to 1 nm (eight atomic layers). In all structures, the ratio of germanium and silicon is protected, while producing interface roughness. Thus, diffusion of Si and Ge by annealing is imitated. Length of the diffusion zone is used as a measure of interface roughness (Figure 4(b)). Figure 4(a) shows that thermal conductivity values are reduced up to 40% along the growth direction. Thermal conductivity is reduced by half via the effect of interface roughness on transverse directions. Less-distributed phonons on transverse directions are directly affected via scattering and these phonons cause more decrease on thermal conductivity compared to growth direction.

Superlattice structures show a dramatic fall in thermal conductivity depending on their symmetry, period and interface roughness, which can be achieved experimentally with a controlled fabrication process. A few experimental studies on thermal conductivity of superlattices also demonstrate the efficient control of thermal conductivity with the superlattice parameters such as interface roughness with controlled growth conditions such MBE. Thus, thermal conductivity could be decreased even below limit of amorphous silicon (1.0 W m${}^{-1}$ K${}^{-1}$) [53]. The lowest thermal conductivity that we achieve with bulk superlattice along growth direction is about 5 W m${}^{-1}$ K${}^{-1}$, which could be decreased to 3 W m${}^{-1}$ K${}^{-1}$ with interface roughness. This value is close to the experimentally observed thermal conductivity of superlattices; the difference could be attributed to the pristine nature of our superlattice layers that do not contain any atomic vacancies, dislocations or atomic impurities.

### 3.2. Cylindrical nanowires

In this section we analyze the one-dimensional cylindrical nanowire structures (c-nanowires). These are alloyed (Si${}_{1-x}$Ge${}_{x}$) c-nanowires, symmetric and asymmetric superlattice c-nanowires.

#### 3.2.1. Alloyed cylindrical nanowires

**Figure 5.**
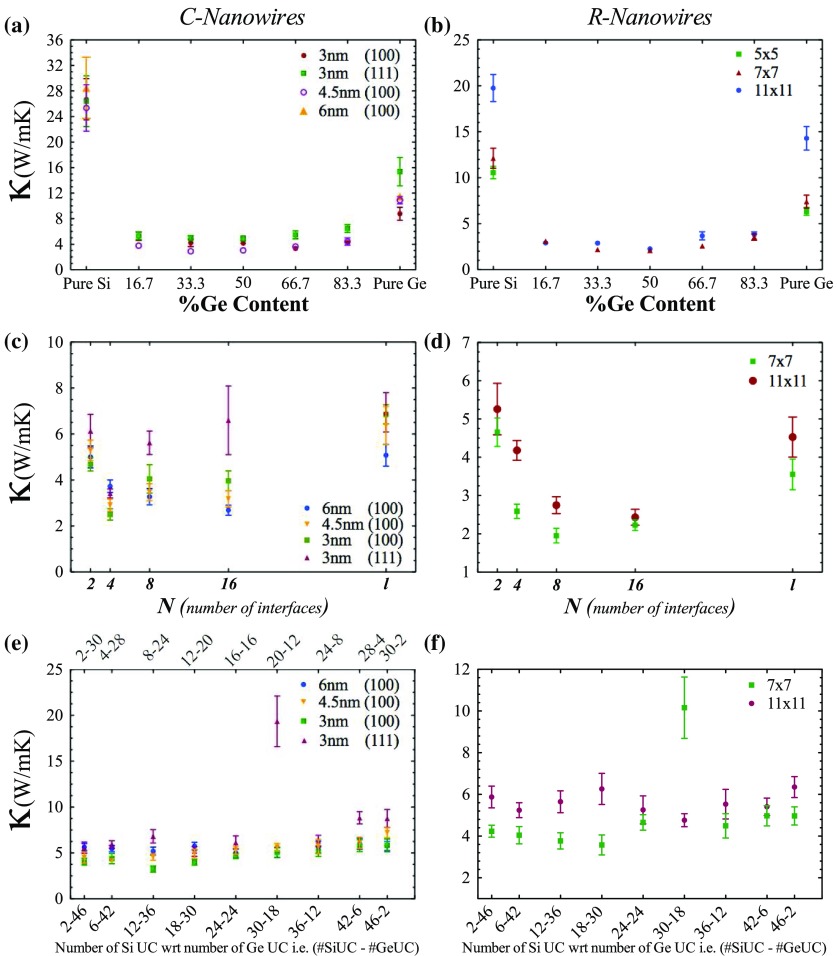
Thermal conductivity values of Si${}_{1-x}$Ge${}_{x}$ (a) cylindrical nanowires with different diameters (d) rectangular nanowires with different cross-section areas. Thermal conductivity values of symmetric (b) cylindrical and (e) rectangular superlattice nanowires with respect to number of interfaces. Thermal conductivity values of asymmetric (c) cylindrical and (f) rectangular superlattice nanowires with respect to numbers of silicon unit cell comparing germanium unit cell.

In order to analyze the effect of diameter and growth direction on the thermal conductivity, we first modeled pure silicon and germanium cylindrical nanowires with diameters of 3.0, 4.5 and 6.0 nm along the <100> growth direction and nanowires with 3.0 nm along the <111> growth direction. Then, we examined the effect of alloying in some of those c-nanowires. Figure 5(a) indicates that diameter affects thermal transport properties due to increased ratio of surface atoms to bulk atoms. Decreasing in diameter or cross section area causes boundary scattering to be enhanced and it has a strong effect on thermal transport [9,18,54]. Furthermore, alloying of c-nanowires decreases the thermal conductivity from 32 to 4 W m${}^{-1}$ K${}^{-1}$ and a small amount of Ge content in Si causes a sharp decrease in thermal conductivity in the system. Local strain has no large contribution on decreasing thermal conductivity, another reason, mass disorder and impurity scattering of phonons is highly responsible for decreasing and achieving minimum thermal conductivity in these systems [26,55]. Minimum thermal conductivity (3.05 W m${}^{-1}$ K${}^{-1}$ for 4.5 nm diameter) is reached at *x* = 0.5 in Si${}_{1-x}$Ge${}_{x}$ c-nanowires, similarly with Chen et al. [55]. NEMD results for phonon participation on thermal transport are lowest at that point.

#### 3.2.2. Superlattice cylindrical nanowires

To accentuate the differences between bulk and low-dimensional superlattices, the c-nanowires used in the alloy case are prepared as superlattice structures and only growth directions are set as periodic. EMD simulations of symmetric cylindrical superlattice nanowires with diameters 3, 4.5 and 6 nm are plotted in Figure 5(b). As mentioned before, superlattices show an anomalous trend and the <111> direction shows a slightly higher thermal conductivity even though its diameter (3 nm) is smaller than the rest of the investigated structures (in Figure 5(b)). Results show that thermal conductivity is first decreased to a minimum *N* value equal to 4 (corresponds to *w* = 12) then increases. This trend cannot be explained by interface scattering at the boundaries. There is a specific length scale in periodicity which demonstrates minimum thermal conductivity in superlattice structures. Phonons have particle and wave characteristics. Minimum thermal conductivity occurs at a transition from wave-dominated to particle-dominated transport [56]. That is the reason why thermal conductivity first decreases then increases with respect to number of interfaces in the system. When the number of interfaces is large enough or equal length value to the unit cell, phonons behaves like waves and show wave-dominated transport. So interface scattering, which disturbs mainly particle-dominated transport, does not show itself directly. Constructive and destructive property of phonon waves decide the thermal conductivity trend in the system. When *N* is small, phonons behaves like a particle but in that time, the number of interfaces is not large enough to have an effect. Therefore, particle-like transport exists in the system. However, these findings indicate that thermal conductivity can be suppressed to 2.5 W m${}^{-1}$ K${}^{-1}$ and even more suppression is possible for other superlattice structures, as demonstrated in Figure 5(e).

Thermal conductivity calculations of asymmetric superlattices of c-nanowires is shown in Figure 5(c). The <111> direction behaves different from the <100> direction. Silicon UC to germanium UC effect in the <111> direction is apparent and TC increases with increase in silicon content but, in the <100> direction, silicon UC to germanium UC change creates a band around 4–5 W m${}^{-1}$ K${}^{-1}$ thermal conductivity value. Small diameters are slightly better in terms of values, as may be seen in Figure 5(c). Thus, a single interface between Si and Ge could effectively improve the thermoelectric performance by decreasing lattice thermal conductivity if the electrical resistance remains intact.

### 3.3. Rectangular nanowires

This section is comprised of two parts: Si${}_{1-x}$Ge${}_{x}$ rectangular nanowire (r-nanowires); and superlattice r-nanowires with periodic boundary conditions set to long axis. Therefore, these wires are considered as one dimensional. Until now, results show that the <100> growth direction gives better thermal resistance, as a candidate for thermoelectrics, compared to the <111> growth direction both in c-nanowire and bulk structures, especially superlattice structures. An experimental report has also shown that the <100> direction has better electrical current characteristics than other directions [57]. Therefore, the <100> growth direction has been selected as a favorable direction for Si-Ge thermoelectrics and that is why <100> growth r-nanowire is preferred in this section.

#### 3.3.1. Rectangular alloyed nanowires

One-dimensional rectangular nanowire alloys are studied in this work. Three structures with same length but different cross-section area are firstly considered. They are respectively, 5×5, 7×7 and 11×11 with 48 UC length. Like the diameter dependency observed in circular cross section (Figure 5(a)), cross-section area dependency exists in r-nanowires (Figure 5(d)). Therefore, size effect one of the important parameter for thermal conductivity reduction is approved. Figure 5(d) shows that thermal conductivity decreases with alloying as found in the c-nanowire case. A small amount of germanium may decrease the thermal conductivity dramatically. Minimum thermal conductivity (2.08–2.25 W m${}^{-1}$ K${}^{-1}$) is reached at nearly *x* = 0.5 in Si${}_{1-x}$Ge${}_{x}$ r-nanowires.

#### 3.3.2. Rectangular superlattice nanowires

To compare 4.5 nm and 6 nm diameter c-nanowires superlattice structures, 7×7 and 11×11 (corresponding same cross section areas, respectively) r-nanowires are selected for superlattice calculations. In Figure 5(e), the thermal conductivity values of symmetric rectangular superlattice nanowires are plotted as a function of periodicity. At room temperature, minimum thermal conductivity in all superlattice structures (including bulk and cylindrical nanowires) is obtained within this section. *w*=6 (7×7) r-superlattice structure presents thermal conductivity values below 2 W m${}^{-1}$ K${}^{-1}$. As noted before in the symmetric part, an anomalous trend is observed. There is a specific periodicity and generally it exits if *N* is 2${}^{n}$ when *n* is 2 (bulk superlattice and superlattice c-nanowire) or *n* is 3 (superlattice r-nanowire). Therefore, investigation of different structures demonstrates that wave to particle transition exist around similar periodicity.

Lastly, an asymmetric case of 7×7 and 11×11-pagination is studied. Again, a band-like trend is observed, ∼ 4 W m${}^{-1}$ K${}^{-1}$ for 7×7 and ∼ 5.5 W m${}^{-1}$ K${}^{-1}$ for 11×11 in Figure 5(f). Interestingly, a peak at 30 Si UC–18 Ge UC was observed. It is clear that thermal resistance at this point is very weak for some structures. According to these results, the rectangular cross section shows lower thermal conductivity than the cylindrical cross section. C-nanowire superlattice show minimum thermal conductivity above 2.0 W m${}^{-1}$ K${}^{-1}$. On the other hand, r-nanowire superlattices can decrease this value below 2.0 W m${}^{-1}$ K${}^{-1}$. Physical interpretation of this dependence can be attributed to the surface to volume ratio (SVR) effect. As noted previously in the literature, for the identical systems (in terms of volume) rectangular nanowires have larger SVR with respect to circular ones [48], and thus have lower lattice thermal conductivity. Therefore, the available area for the phonon scattering increases and results in lower TC in rectangular nanowires. Therefore, superlattices in the form of nanowire architectures are one of the best candidates for the thermoelectric applications in terms of their low lattice thermal conductivity at room temperature and higher temperatures. Although not within the scope of this study, this low lattice thermal conductivity can be translated to a higher *ZT* if electrical conductivity is at least not decreased significantly. Ab initio and experimental studies are encouraging. For example, ab initio calculations on ultrathin superlattice nanowires indicate no significant change in the electrical conductivity [42,58]. The experimental Seebeck value of a Bi${}_{2}$Te${}_{3}$/Sb${}_{2}$Te${}_{3}$ superlattice is close to that of a state-of-the-art bulk Bi${}_{2}$Te${}_{3}$/Sb${}_{2}$Te${}_{3}$ alloy, resulting in a *ZT* ∼ 2.4 for the superlattice owing to its ultralow thermal conductivity [59]. Epitaxially grown nanocrystal superlattices also demonstrate superior electrical conductivities due to the coherence and alignment between nanocrystals [60,61].

**Figure 6.**
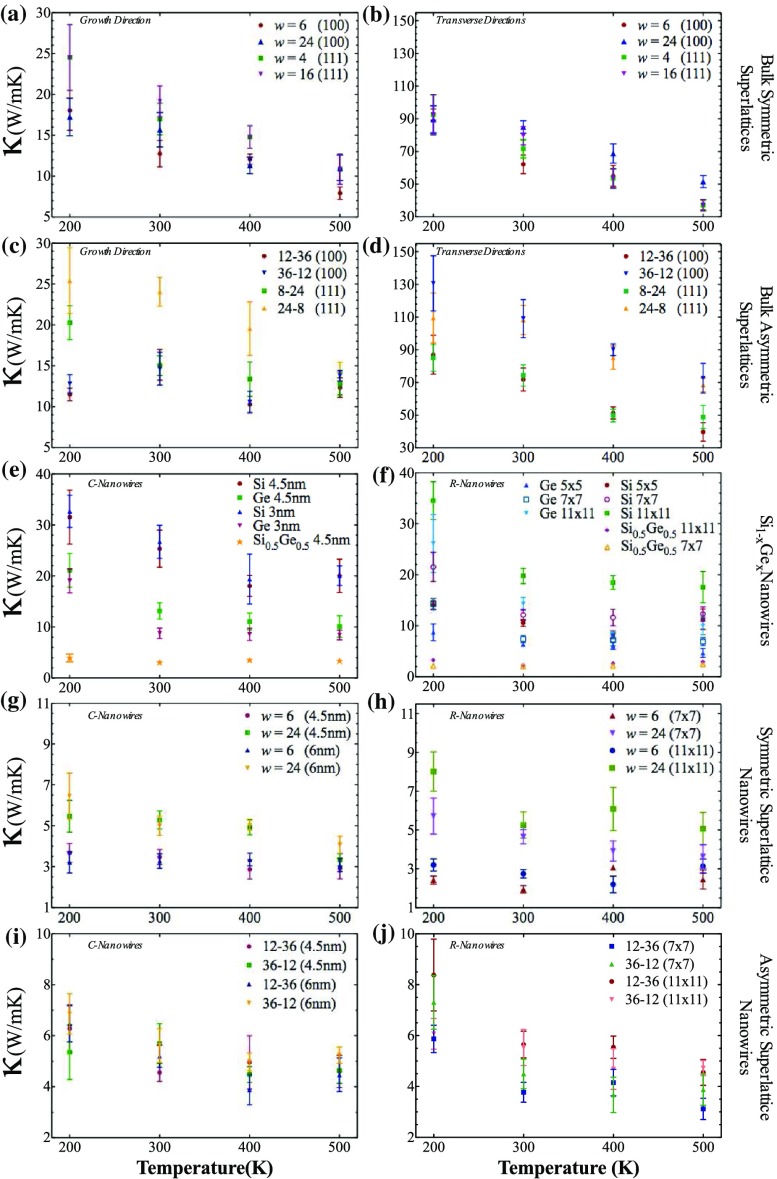
Thermal conductivity analysis of selected structures with respect to temperature.

### 3.4. Effect of temperature

To discover details of temperature effect on thermal transport properties of Si-Ge systems, some of the selected structures are investigated. The thermal conductivity of the selected structures are computed for different temperatures, 200, 300, 400 and 500 K (Figure 6). Figure 6(a) and (b) show the variation in thermal conductivity at temperatures between 200 and 500 K alongthe growth and transverse directions of selected bulk symmetric superlattices, while Figure 6(c) and (d) show the corresponding values for selected bulk asymmetric superlattices. Figure 6(c), (g) and (i) show thermal conductivity at 200–500 K for alloyed c-nanowires, symmetric and asymmetric superlattice c-nanowires, respectively, while Figure 6(f), (h) and (j) show the corresponding values for alloyed r-nanowires. The nanowires are labeled in the top-left corner of each panel and in the right part of the figure.

Two dominant trends are observed in temperature effect on thermal transport calculations for Si-Ge structures. The former is thermal conductivity decreases while temperature increases, as expected due to increase in phonon–phonon interaction and Umklapp scattering. This general trend can be seen nearly in whole structures. The latter, thermal conductivity is independent from temperature changing between 200 and 500 K, can be observed only structures in which thermal conductivity is too much suppressed or, phonons movement or participation are restricted. For instance, in Figure 6(e) and (f), *x* = 0.5 in Si${}_{1-x}$Ge${}_{x}$ c-nanowires and r-nanowires. Another example is *w* = 6 in Figure 6(g) and (h), corresponding to symmetric superlattice c-nanowires and r-nanowires, respectively.

## 4. Conclusions

In summary, systematic equilibrium molecular dynamics simulations are performed with the Tersoff interatomic potential to investigate thermal conductivity of bulk and one-dimensional structures. Lattice thermal conductivity of one-dimensional structures are remarkably lower than the bulk structures. Reduction of the thermal conductivity of the one-dimensional structures with nanostructuring is also studied in this work to provide additional strategies to increase thermoelectric performance such as incorporation of one single interface, a rough interface, alloying or producing a superlattice system. In terms of thermal conductivity values, comparison between superlattice and alloy nanostructures shows negligible difference. However, superlattices that have a particular periodicity show a lower thermal conductivity (<2 W m${}^{-1}$ K${}^{-1}$) than other structures such as alloys (>2 W m${}^{-1}$ K${}^{-1}$). In terms of experimental realization, alloying with nanostructuring could be a more straightforward method than fabrication of superlattice nanowires to decrease lattice thermal conductivity. The trade-off between two structures (alloy or superlattice) could be evaluated further by considering electrical resistivity. We conclude that superlattice structures are better thermoelectric candidates due to the fact that their electrical conductivity or Seebeck coefficient is not deteriorated for the optimized systems, as explained in the discussion. Within the scope of this study, we show that some superlattice nanostructures such as rectangular superlattice nanowires are appropriate for high-performance thermoelectric materials. Our results clearly point out the possible lattice thermal conductivity tune mechanisms with nanostructure engineering.
